# Reflections on the role of opioids in the treatment of chronic pain: a shared solution for prescription opioid abuse and pain

**DOI:** 10.1111/joim.12345

**Published:** 2015-02-02

**Authors:** D. Thomas, J. Frascella, T. Hall, W. Smith, W. Compton, W. Koroshetz, J. Briggs, P. Grady, M. Somerman, N. Volkow

**Affiliations:** ^1^ Division of Clinical Neuroscience and Behavioral ResearchNational Institute on Drug AbuseBethesdaMDUSA; ^2^ Office of Behavior and Social Sciences ResearchNational Institutes of HealthBethesdaMDUSA; ^3^ Office of the DirectorNational Institute on Drug AbuseBethesdaMDUSA; ^4^ Office of the DirectorNational Institute of Neurological Disorders and StrokeBethesdaMDUSA; ^5^ Office of the DirectorNational Center for Complementary and Integrative HealthBethesdaMDUSA; ^6^ Office of the DirectorNational Institute of Nursing ResearchBethesdaMDUSA; ^7^ Office of the DirectorNational Institute of Dental and Craniofacial ResearchBethesdaMDUSA

‘The Role of Opioids in the Treatment of Chronic Pain’ was the title of a recent workshop (September 29–30, 2014) supported by the National Institutes of Health (NIH) Office of Disease Prevention that highlights the relevance and urgency of the topic. High‐quality research presently does not exist to definitively answer questions about opioid safety, efficacy and abuse potential with long‐term use 1. However, prescriptions for opioids increased from 76 million in 1991 to 207 million in 2013, which were associated with parallel increases in opioid‐related morbidity and mortality 2. Currently, in the U.S., close to 100 million Americans suffer from chronic pain and may be given prescription opioids, along with close to two million Americans who abuse opioid analgesics 3, with over 16 000 overdose deaths attributed to prescription opioids 4. This situation reflects both the challenge to effectively treat the complex condition of chronic pain and the lack of understanding about opioid abuse potential and the risk of addiction in healthcare settings.

The medical needs of so many people in chronic pain overwhelm much of our healthcare system, and this problem continues to worsen. Inadequate provider education of how to treat chronic pain compounds the problem. U.S. medical students get a median of only 9 h of training that relate in some way to pain over their 4 years of medical school 5, resulting in inadequate training for many physicians on the effective use of opioids. This is further compounded by the minimal training that medical students get regarding substance use disorders 6, resulting in many of them being unprepared to recognize and monitor risks or signs of addiction in their patients. Furthermore, the structure of the American healthcare system may hinder access to adequate pain management. Opioids are frequently used as the first and only option for treating chronic pain, which is partly explained by a medical treatment reimbursement system that generally does not cover the multimodal treatment approaches for effective management of chronic pain 7.

As the Pathways to Prevention workshop report identifies, there is a necessity to focus on developing a rigorous research base to inform clinical and policy decision‐making. Research funders and pharma should increase support for studies to establish better data on the utility of opioids in the treatment of chronic pain. Specifically, studies are needed to understand the efficacy of long‐term opioid use in the treatment of chronic pain; efficacy of opioid treatment for individuals who might have had prior problems with prescription opioids or other drugs of abuse, and what might be the best treatment course for this population; the potential for misuse, abuse and addiction in the long‐term use of opioids for pain; and potential individual differences that could predict who might respond best to which treatments (e.g. pharmacogenetics, pain/drug history, pain subtypes, length of chronic pain).

Whilst providing funding for additional research is one strategy, the NIH (and the National Institute on Drug Abuse, in particular) also supports additional approaches to reducing opioid misuse, abuse and addiction. Expanding curriculum for education on chronic pain management in medical, nursing, pharmacy, dental and other professional schools is being addressed by the NIH Pain Consortium (a multi‐institute consortium that deals with pain issues across the NIH) through the Centers of Excellence in Pain Education programme (http://painconsortium.nih.gov/NIH_Pain_Programs/CoEPES.html).

Another strategy for an effective pain management focuses on the development and use of nonopioid pain medications, complementary and alternative medical treatments, other medications, physical therapy, stress management and cognitive/behavioural therapy 8. Systematic reviews of acupuncture have revealed benefits for pain management in postoperative patients and in tension headache treatment 9, 10. There is also evidence that cognitive‐behavioural therapy helped individuals with chronic pain 11, 12. A meta‐analysis of interdisciplinary pain management programmes that included physical therapy showed both intensity of pain and consumption of pain medication decreased significantly 13. These options may offer pain patients treatments that could improve pain, function and quality of life and decrease the reliance on opioid medications.

More comprehensive systems for treating pain are needed, for the current options fail to deliver significant relief for individuals with severe chronic pain 7. Dersh *et al*. 14 concluded that treatments without a biopsychosocial approach will most likely put a great number of chronic pain patients at a higher risk for prolonged disability. Existing data on multidisciplinary pain management show consistently effective benefits for chronic back pain relief that exceed surgical results, enabling patients to return to work and live more active and productive lives 15. Yet, these more integrative and individualized therapies may not be reimbursed by health insurance, making them less accessible to patients 7.

Importantly, individualized care for chronic pain may still involve prescription opioids. Because their role will vary from patient to patient, potentially changing during the scope of treatment, individualized therapy for chronic pain should also include careful prescription drug monitoring. Especially when integrated into electronic healthcare records, such information will help healthcare providers better detect opioid misuse, abuse and diversion. Including prescription drug monitoring in individualized treatment for chronic pain could help identify those struggling with addiction and lead to their treatment for this problem. More careful monitoring can also aid in determining exactly what treatments for pain management an individual receives, and what works for that patient. The provider can then use this information to adjust individualized treatment plans for chronic pain.

Even with careful monitoring of prescription opioid use, overdose may still occur with misuse, abuse and also with ordinary use of opioids in pain patients. Therefore, strategies that minimize the risks of overdose should be emphasized as well. For example, naloxone, an opioid antagonist that can reverse the life‐threatening effects of an overdose, saves lives when made more accessible 16. As overdoses are more likely to occur in patients that receive higher doses of opioid medication and in those using benzodiazepines, special care should be given to properly monitor these patients and educate their relatives or caregivers on the proper use of naloxone.

In summary, a critical need exists for better and more comprehensive chronic pain treatment in the United States. Required research must determine the efficacy of various treatment options for chronic pain, including the role of opioids. In addition to having an in‐depth understanding of the physical nature of this condition, healthcare professionals treating chronic pain should also have a keen clinical understanding of the psychosocial, emotional and affective aspects of this condition. Furthermore, treatment systems may need to be modified to make available the intensive, interprofessional/integrative and much more individualized treatment requirements of the most complex of these patients.

The growing numbers of patients with chronic pain, along with the increases in the abuse and overdoses from prescription opioids in the U.S., highlight the urgency to instate better education for healthcare providers in the management of chronic pain and addiction, but also the need to develop better systems for managing and treating chronic pain.

## Conflict of interest statement

No conflict of interest was declared.
